# Development of a benchmark tool for cancer centers; results from a pilot exercise

**DOI:** 10.1186/s12913-018-3574-z

**Published:** 2018-10-10

**Authors:** Anke Wind, Joris van Dijk, Isabelle Nefkens, Wineke van Lent, Péter Nagy, Ernestas Janulionis, Tuula Helander, Francisco Rocha-Goncalves, Wim van Harten

**Affiliations:** 1 The Netherlands Cancer Institute-Antoni van Leeuwenhoek Hospital, Amsterdam, the Netherlands; 2 Department of Health Technology and Services Research, University of Twente, P.O. Box 217, 7500 AE Enschede, The Netherlands; 3 PANAXEA, Amsterdam, the Netherlands; 4 OLVG, Amsterdam, the Netherlands; 5 Department of Molecular Immunology and Toxicology, National Institute of Oncology, Budapest, Hungary; 6 National Cancer Institute, Vilnius, Lithuania; 7 Comprehensive Cancer Center, Helsinki University Hospital, and University of Helsinki, Helsinki, Finland; 8 Portuguese Institute of Oncology Porto (IPO-Porto), Porto, Portugal; 9 Rijnstate Hospital, Arnhem, the Netherlands

**Keywords:** Benchmarking, Quality of care, Quality improvement, Cancer centers

## Abstract

**Background:**

Differences in cancer survival exist between countries in Europe. Benchmarking of good practices can assist cancer centers to improve their services aiming for reduced inequalities. The aim of the BENCH-CAN project was to develop a cancer care benchmark tool, identify performance differences and yield good practice examples, contributing to improving the quality of interdisciplinary care. This paper describes the development of this benchmark tool and its validation in cancer centers throughout Europe.

**Methods:**

A benchmark tool was developed and executed according to a 13 step benchmarking process. Indicator selection was based on literature, existing accreditation systems, and expert opinions. A final format was tested in eight cancer centers. Center visits by a team of minimally 3 persons, including a patient representative, were performed to verify information, grasp context and check on additional questions (through semi-structured interviews). Based on the visits, the benchmark methodology identified opportunities for improvement.

**Results:**

The final tool existed of 61 qualitative and 141 quantitative indicators, which were structured in an evaluative framework. Data from all eight participating centers showed inter-organization variability on many indicators, such as bed utilization and provision of survivorship care. Subsequently, improvement suggestions for centers were made; 85% of which were agreed upon.

**Conclusion:**

A benchmarking tool for cancer centers was successfully developed and tested and is available in an open format. The tool allows comparison of inter-organizational performance. Improvement opportunities were successfully identified for every center involved and the tool was positively evaluated.

**Electronic supplementary material:**

The online version of this article (10.1186/s12913-018-3574-z) contains supplementary material, which is available to authorized users.

## Background

The number of cancer patients is steadily increasing and, despite rapid improvements in therapeutic options, inequalities in access to quality cancer care and thus survival exist between different countries [1]. These inequalities indicate room for improvement in quality of cancer care, identifying good practices can assist cancer centers(CC’s) in improving their services and can ultimately reduce inequalities, benchmarking is an effective method for measuring and analyzing performance and its underlying organizational practices [2]. Developed in industry in the 1930s, benchmarking made its first appearance in healthcare in 1990 [2]. Benchmarking involves a comparison of performance in order to identify, introduce, and sustain good practices, this is achieved by collecting, measuring and evaluating data to establish a target performance level, a benchmark [3]. This performance standard can then be used to evaluate the current performance by comparing it to other organizations, including good-practice facilities [3]. Due to globalization, absence of national-comparators, and the search for competitive alternatives, there is an increasing interest in international benchmarking [4]. However, a study by Longbottom [5] on 560 healthcare benchmarking projects, showed only 4% of the projects involved institutions from different countries. In literature, relatively few papers are published on healthcare benchmarking methods [6]. Moreover, to the best of our knowledge, there is no confirmed indicator set for benchmarking comprehensive cancer care. In 2013, the Organization of European Cancer Institute (OECI) [7] therefore launched the BENCH-CAN project [8], aiming at reducing health inequalities in cancer care in Europe and improving interdisciplinary comprehensive cancer care by yielding good practice examples. In view of this aim, a comprehensive international benchmarking tool was developed covering all relevant care related and organizational fields. In this study comprehensive refers to thorough, broad, including all relevant aspects - which is also a means to describe interdisciplinary, state of the art, holistic cancer care. In line with the aim of the BENCH-CAN project, the objectives of this study were (i) to develop and pilot a benchmark tool for cancer care with both qualitative and quantitative indicators, (ii) identify performance differences between cancer centers, and (iii) identify improvement opportunities.

## Method

### Study design and sample

This multi-center benchmarking study involved eight cancer centers (CCs) in Europe, six of which designated as a comprehensive cancer center (encompassing care, research and education) by the OECI [9]. A mix of geographic selection and convenience sampling was used to select the pilot sites. Centers were chosen based on national location, in order to have a good distribution between geographical regions in Europe and secondly willingness to participate. All centers had to be sufficiently organized and dedicated to oncology, and treat significant numbers of cancer patients. Centers were located in three geographical clusters: North/Western-Europe (*n* = 2), Southern-Europe (*n* = 3) and Central/Eastern-Europe (*n* = 3). The benchmark tool was developed and executed according to the 13-step method by van Lent et al., [6] (see Table 1). In short, the first five steps involve the identification of the problem, forming the benchmarking team, choosing benchmark partners and define their main characteristics, and identify the relevant stakeholders. Step 6 to 12 will be explained in more detail in the following paragraphs. Ethical consideration was not applicable in this study.

**Table 1:** Benchmarking steps developed by van Lent and application in this study

13 steps by van Lent	Application of the steps in this study
1 Determine what to benchmark	Comprehensive cancer care, structured through the domains of the BENCH-CAN framework such as People, Process, Product & Services, and Efficient (step 6).
2 Form a benchmarking team	International consortium existing of representatives from cancer centers, health R&D organisation, biomedical innovations consultancy company, and OECI.
3 Choose benchmarking partners	Cancer centers in Europe.
4 Define and verify the main characteristics of the partners	A mapping exercise of the external environment in which the cancer centers are located was performed.
5 Identify stakeholders	Four stakeholder groups were identified: patients, management, clinicians and researchers.
6 Construct a framework to structure the indicators	The framework is based on the European Foundation for Quality Management (EFQM) Excellence Model [10] and the adapted six domains of quality of the Institute of Medicine [13].
7 Develop relevant and comparable indicators	Indicators were retrieved from literature [14] and expert opinion.
8 Stakeholders select indicators	Stakeholders from the BENCH-CAN project and other experts from cancer centers provided feedback on the indicators.
9 Measure the set of performance indicators	Indicators were first pre-piloted in three centers to check clarity of the definitions and whether indicators would yield interesting information. Data collection phase was three months. Next, the three month during data collection phase was repeated for the other centers. A team performed a center visit to each pilot center to verify the data, to grasp the context and clarify any questions arising from the provided data.
10 Analyse performance indicators	The researchers compared the performance of the pilot cancer centers. Reports of this comparison were checked by the other members of the center visit team.
11 Take action: results are presented in a report and recommendations are given	For each participating cancer centre, a report was made containing the outcomes of the benchmark for all centers. Data was anonymized. Improvement recommendations were sent in a separate document.
12 Develop relevant plans	Pilot centers were asked to develop improvement plans for recommendations that they agreed with.
13 Implement the improvement plans	Outside the scope of this study.

### Framework and indicators

**Fig. 1:**
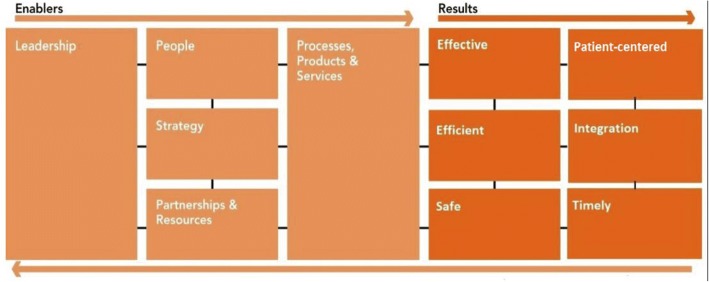
the BENCH-CAN framework. Note: The enabler domains from the EFQM model describe factors that enable good quality care. The results domains adapted from the IOM domains of quality describe how good quality care can be measured

As described in step 6 we developed a framework to structure the indicators. The European Foundation for Quality Management (EFQM) [10] Excellence Model (comparable to the Baldridge model [11]) was used for performance-assessment and identification of key strengths and improvement areas [12]. Apart from the enabler fields, we adapted the Institute of Medicine domains of quality [13] for outcomes or results: effective, efficient, safe, patient-centered, integration and timely (Fig. 1).

Indicators (step 7) were derived from literature [14] and expert opinion. Existing assessments were used as basis for the benchmark tool [15]. Stakeholders of the BENCH-CAN project such as representatives from the European Cancer Patient Coalition (ECPC), and clinicians and experts (such as quality managers) from cancer centers (OECI member centers, *n* = 71) provided feedback to reach consensus on the final set of indicators to be used in the benchmark (step 8). As one person per center was asked to collect feedback within that specific center, it cannot be determined whether the feedback was shared equally by the different stakeholder groups. The combination of data provision, site visit by a combined team and feedback provided sufficient possibilities for cross checking. For the financial and quantitative indicators this included the standardization of data collection to allow comparison between pilot centers and determining the level of detail for cost accounting.

### Reliability and validity

A priori stakeholder involvement was used to ensure reliability and validity [6]. After collecting the indicators in step 9, the validity of the indicators was checked using feedback from the pilot centers based on three criteria [16, 17]: 1) definition clarity, 2) data availability and reliability, 3) discriminatory features and usability for comparisons.

### Indicator refinement and measurement

The indicators were pre-piloted in three centers to see whether the definitions were clear and the indicators would yield relevant, discriminative information. These three centers were selected based on willingness to participate and readiness to provide the data in a short period. Based on this pilot, we decided to add and remove indicators, and refine definitions of some indicators. After refinement, the resulting set of 63 qualitative indicators and 193 quantitative indicators was measured in the five remaining centers. The pre-pilot centers submitted additional information on the added indicators in order to make all centers comparable.

We collected data from the year 2012 and each pilot center appointed a contact person who was responsible for the data collection within the institute and the delivery of the data to the research team. After a quick data scan, a one-day visit to each pilot center was performed to verify the data, grasp the context and clarify questions arising from the provided data. The visits were performed by the lead researcher, a representative from the ECPC and representatives of (other) members of the consortium. The visits were also used to collect additional information through semi-structured interviews and to acquire feedback on the benchmark tool. In the semi-structured interview, the lead researcher provided some structure based on the questions that arose from the quick scan (see Additional file 1: Appendix 1 for a selection of five topics and corresponding questions in the semi-structured interviews) but worked flexibly and allowed room for the respondent’s more spontaneous descriptions and narratives and questions from the other site visit members [18].

### Analysis

Two methods were used to compare the qualitative and quantitative data. A deductive form of the Qualitative Content Analysis was used to analyze the qualitative data [18]. This method contains eight steps which are described in Table 2.

**Table 2:** steps Qualitative Content Analysis [26]

Step	Action
1	Read through the benchmark data (transcripts) and make notes
2	Go through the notes and list the different types of information found
3	Read through the list and categorize each item (domains of the framework were used as main categories)
4	Repeat the first three stages again for each data transcript
5	Collect all of the categories or themes and examine each in detail and consider it’s fit and its relevance
6	Categorize all data (all transcripts together) into minor and major categories/themes
7	Review all categories and ascertain whether some categories can be merged or sub-categorized
8	Return to original transcripts and ensure that all the information has been categorized

Quantitative data was first checked for consistency and correctness, and all cost data was converted into euros and adjusted for Purchasing Power Parity [19]. In addition, data was normalized when necessary to be able to compare different types and sizes of centers. Used normalizations were: 1) openings hours of departments, 2) number of inpatient beds, 3) number of inpatient visits, and 4) number of full-time equivalent (FTE). All data was summarized and possible outliers were identified. Outliers were discussed with the relevant centers to elaborate on the possible reasons for the scores.

To ensure validity, a report with all data (qualitative and quantitative) was send to the pilot centers for verification. Not all centers were able to provide all data, as some were not able to retrieve and produce the data and others were concerned with the time needed to gather all the requested information. Hence, for some indicators centers are missing, as we did not use imputation. Data is structured according to the adapted domains of quality from the IOM; effective, efficient, safe, patient-centered, and timely.

### Improvement suggestions

After comparison of all quantitative and qualitative data, three researchers independently identified improvement opportunities for each center. Improvement suggestions or opportunities (at least three per center) were only mentioned for those areas where the researchers felt the center could actually make the improvement without being restricted by for example regulations. Based on these improvement suggestions, if in agreement, pilot centers developed improvement plans.

## Results

### Reliability and validity

Ten indicators deemed irrelevant (such as sick leave) were removed after the pre-pilot. Nineteen indicators were added based on evaluation criteria and feedback. Several indicator definitions were clarified. The final pilot-list contained 63 qualitative indicators and 193 quantitative indicators. After the pilot data collection, a secondary evaluation of the definition clarity, data availability, data reliability and discriminative value was performed. This re-valuation resulted in a final set of 61 qualitative indicators and 141 quantitative indicators that were deemed suitable for wider use in benchmarking cancer centers (Additional file 2: Appendix 2).

### Performance differences between centers

The performances of the participating centers varied on many indicators, of which a selection is shown in Table 3 and described below. Organizations are anonymized. The results are structured according to the adapted domains of quality [13].

**Table 3:** Profiles of the cancer centers against a selection of indicators. For each domain a selection of indicators and their outcomes is presented

Topic	Center							
	A	B	C	D	E	F	G	H
Type of center	Comprehensive CC	Comprehensive CC	Clinical CC	Comprehensive CC	x	Comprehensive CC	Comprehensive CC	Comprehensive CC
Effective								
Survival/mortality registration	Gross mortality ratios (2.08%)per tumor type and -stage are published on the website.	Gross mortality ratio per number of discharges is registered (9,5%) and Risk-Adjusted Mortality (e.g.age, sex, tumor etc.) (0,8836)	Gross mortality rate is registered	Gross inpatients mortality ratio (2.0%) is registered.	Gross mortality ratio is registered	Mortality ratios are collected only for patients who had surgery	Mortality ratios are registered by the National Cancer Registry, published on the website.	Not recorded at institutional level
Colo-rectal surgery mortality (within 30 days)	0	Not registered	Not registered	0	Unknown	Unknown	Not registered	Unknown
Innovative technology and therapies	Budgets are not keeping up with the developments in technology’s and therapies and the increase in costs that comes with this	Targeted biological treatments are not covered. New therapies will lead to a negative balance. This will cause a big challenge in the future.	It is hard to introduce the latest technologies because they are not reimbursed. It might take 2–3 years to arrange reimbursement.	- New treatments are very expensive, not always paid for by insurance - Healthcare services are not able to provide state of the art treatment to all patients	Not foreseen as a major challenge.	Biosimilar, and generics are a challenge. Several expensive drugs come to the market. Need for biomarkers	All drugs are reimbursed, but there is a 6 month wait before the reimbursement comes. The national system is slower. ESMO guidelines are followed.	Unknown
Safe								
Risk management	- Quality, Occupational health and environment service - Prospective risk assessments by MDT - Staff training - Dedicated emergency managers, available at the institute 24/7.	- Occupational health and Overall Risk Management Service - Risk management system: general risk management and clinical risk management	- Annual risk factors analysis and prevention action plan. - Evaluation of implementation end each year.	- Several protocols for risk management - New employees undergo a thorough medical examination to decide if they are fit for employment. - Emergency Assessments - External Risk Assessments	-Department for prevention and control of nosocomial infections. - Staff involved is formally trained and informed about the regulations	- Health and Safety function deals with prevention and management of staff safety - The medical physics unit manages the prevention and control of radioactive risks for patients and staff.	- Strategy and program measurements are part of the institute and available on the website	- Risk management plan overseeing clinical management and risk -Medical Directorate coordinates all activities of preventive medicine and environmental health - Protection and Prevention Service.
Adverse events	- Safe incident reporting system - Every employee of the institute has access to this system and can report incidents - Incidents are analyzed by relevant departments and feedback is given to the detector and an advice for improvement measures to the manager - Patient safety committee monitors hospital-wide	- Everybody is allowed to make a notification of an adverse event - Form allows notifier to make recommendations to prevent this situation - All adverse events are collected by the CG service - Quarterly reports on website	- Every staff member can inform the head of clinical department about potential threats. - Medical staff fill a Report of discrepancies or adverse event and inform Head department - Risk and prevention factors evaluation group prepare annual Risk factors and prevention actions plan for the Institute	- Adverse events and near misses reported on departmental level and institutional level - Radiotherapy department has an IT system for reporting irregularities and adverse events - Analysis of irregularities is reported on the shared drive and as well as patient complaints analysis	- Patient incidents are reported to the complaints registry thereafter addressed to the Management, and after evaluation addressed to the Ethics Council. - Every incident is electronically recorded and sent to the public health authorities quarterly. - In every department there is a local registry for incidents, reporting on paper. - Medical errors: if nurse is involved she reports it to the doctor, if the doctor finds it serious, sends it to the medical committee. Nurses cannot report an incident directly.	- Institutional Incident Reporting program. The events are reported on a voluntary and not-anonymous base; - Summary report is annually shared with the strategic directorate. - Reporting usually on paper, but some departments have it electronically - Patients can report incidents as well	System that registers and generates reports for patient satisfaction, patient safety, patient complaints.	- The “incident reporting module” is available for staff in the institutional intranet and a counseling service is available. - Sheets are anonymous. - In practice only the actual events are reported.
Patient centered								
Case manager	Case managers for head and neck cancer, breast cancer and melanoma - Case manager usually nurse specialist or physician assistant - Contact person for questions about their treatment etc. - Case managers redirects patients to supportive services - All patient receive leaflet with all information on how to reach their case manager.	The patient can contact: The physician at an appointment; - A member from the nursing staff that follows the patient; - The social worker and/ or the psychologist that follows the patient - An administrative office that is called “Patient Support Office”	- Patients are informed by treating physicians - Nurses are also indicated as contact persons	- Contact person for each patient, by law, is their responsible specialized MD - a designated Case Manager exists at the Same Day Surgery Unit - Tasks of the Case Manager: - Schedule procedure and inform patient in writing - Collect the necessary paperwork and provide them to the anesthesiologist - Provide patients with discharge records	The contact person for each patient is the physician (medical oncologist, surgeon etc.). The discharge letter contains all the information the patient needs, including a telephone number for the doctor in charge of the case, and in some cases, the number of the nurse of the day hospital clinic.	There is a contact person/team – usually nurses - for each person. A contact number and all the needed pieces of information are given to the patient before discharge. In the breast unit there is one case manager to act as a contact person.	- “personal support nurse” there in practice, not officially - when needed there is option contact physician in charge	Basically there is a physician as contact person for patients but a specific case manager is not appointed for each patient. In clinical trials a case manager, often a research nurse, is appointed to coordinate appointments (exams, visits, follow-up) as primary contact for patients.
Patient involve-ment (care)	- Patient portal: access to their full medical file - Patients can choose their own doctor - Patient participation in decision-making regarding treatment depends on type of health insurance. Not all treatments covered by all types of health insurance	- Patients participate in the multidisciplinary appointments - Physician discusses alternative treatments with the patient - Patients can choose their own doctor	- Patients receive information about their diagnostic and treatment processes and must sign Statement of Faith. - Information to patients and other entities is provided according to procedure - Patients can choose their own doctor	- Patients can choose their own doctor - Patients have the right to a Patient Representative - Patients have the right to review their patient documentation and pose any questions	- Patients are explained the purpose of every diagnostic or therapeutic procedure and alternatives where available. The patient signs informed consent forms at every major step of the care pathway. - Patients have the right to review their patient documentation and pose any questions - Patients can choose their own doctor	- Oncologist and/or the multidisciplinary team propose the diagnostic and treatment processes. If the patient decides to accept the proposal, he/she has to sign the informed consent. -Patients have the right to review their patient documentation and pose any questions	- MDT makes a recommendation, and physician decides with the patient. Patient receives most of the patient documentation automatically (and largely in e-format). - All patients (citizens) have access to their own patient records	- Patients are involved in the discussion of their diagnostic and treatment plan during visits performed by physicians. Signature of the informed consent is intended as the acceptance of the patient to the proposed treatment.
Patient involvement (strategy)	- Patients are represented by the Patient Advisory Board (PAB). - PAB promotes the common interests of patients and gives solicited and unsolicited advice to the Board of Directors	- Patient Support Office to establish connection between users and Board and streamline communication of the patient and the professionals	- Patient representatives are not involved that much due to the lack of legislation and to the fact that representatives are not very active.	- The Patient Representative participates in the Board of Directors’ meeting each week and communicates directly with the Board.	- Patients can participate in the institute through offering suggestions or filing complaints/suggestions during their care in the institute.	- Patients’ representatives are part of the Patient Education working group and proactively propose improvements to services	- For some brochures patients are asked for opinion on design. - Patient Advisory Board, heavily involved in developing services and care. - There is a good relationship with the national Cancer Society.	- Collaboration with external patient organizations that address patients’ priorities and needs.
Survivors	- The institute offers multidisciplinary rehabilitation - General rehabilitation and special rehabilitation program for patients with head and neck cancer. - % Annual budget for survivorship programmes: 0.6%	- Website under development: “I Have Cancer” -Groups related with the institute that give support to patients. - % Annual budget for survivorship programmes: 0	-Rehabilitation treatment -Cancer Information Center -Patient’s school -Social services and other rehabilitation services. - % Annual budget for survivorship programmes: 0	- The survivorship programs are organized in collaboration with the League Against Cancer. - % Annual budget for survivorship programmes: 0	- Psychological support is available in the Institute for patients and their families. - Follow up program for survivors - % Annual budget for survivorship programmes: 0	- The institute has a specific clinic for long term cancer survivors and cancer-free patients and a dedicated patient organization. - % Annual budget for survivorship programmes: 0	- Support unit. - Physiotherapy, Psychosocial support, Nutrition, Peer support, Sexuality, social support, support for families with children - % Annual budget for survivorship programmes: 0.4%	- Chaplain and/or social workers and/or psychologists -Multidisciplinary collaborations to support cancer patients and survivors - % Annual budget for survivorship programmes: 0
Timely								
Waiting and throughput registration	- Access and throughput standards (maximum times) set by government - Dashboard quality and safety reports quarterly on waiting times - Waiting times published on website - Pass standards entry level requirement for negotiations with insurance companies	- Maximum set by government. - Reported in annual report	- Maximum waiting times - Waiting times are available in Institute information board.	- Waiting times are kept according to government decree	- Maximum set by government. - There are currently no standard means of measuring waiting and throughput times.	- The center records waiting times at two institutional levels: 1- Regional: 2- National: Ministry of Health oversight.	-Waiting times on the website for several tumors: breast, prostate and bowel cancers (more to be added).	- Waiting and throughput times are recorded and published on website, -Regional level maximum waiting times. -No consequences regarding reimbursement.
Average overall waiting time before first visit	9.1 days	1.54 days	12 days	7 days	not available	21.8 days	Unknown	9.6 days
Average waiting time from first visit to diagnosis	9.1 days.	Majority patients already have a diagnosis when they come to the first visit.	20 days	14 days	Average not available, depends on pathology result (availability for pathology result is established by law and is maximum 30 days).	Not available	Not available.	Not available
Average waiting time diagnosis-start treatment	Not available	6.53 days	Not available	7 days	Not available	19.7 days	14 days	12.6 days

#### Effective

The majority of centers register crude mortality rates of their patient groups (*n* = 6) as shown in Table 3. Only Institute A publishes this rate. Another type of mortality, 30-day surgical mortality, was not registered in center B, C and G. Centers also reported difficulties with providing novel technologies and therapies limiting their ability to provide the optimal care for patients.

#### Efficient

##### Medical efficiency

**Fig. 2:**
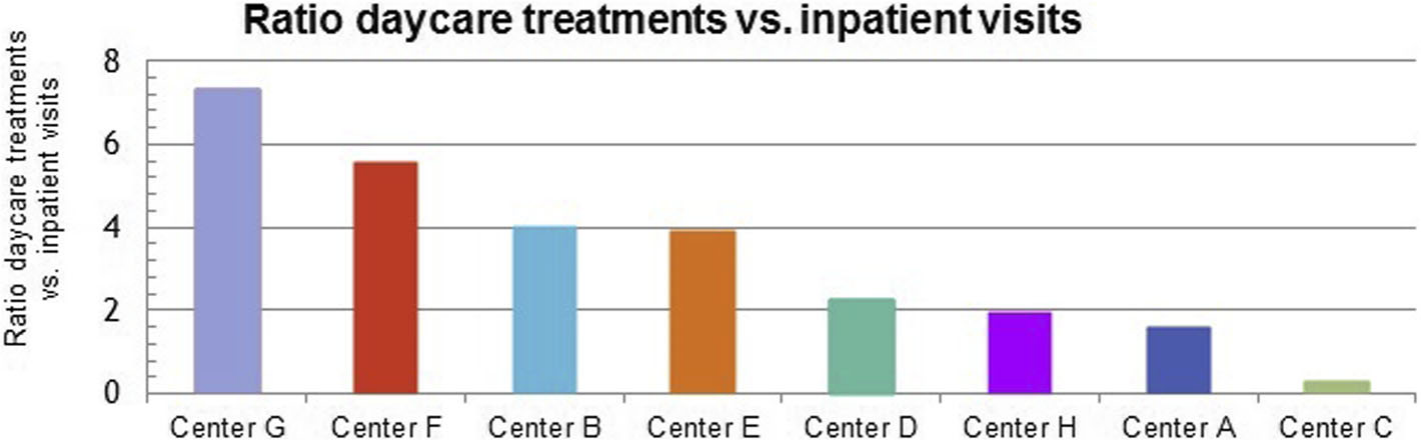
Number of daycare treatments in relation to the number of inpatient visits

The medical efficiency, defined as the use of medical production factors to gain desired health outcome with a minimum waste of time, effort, or skills, greatly varies between the participating centers as shown in Fig. 2. Center G scores high (ratio of 7), whereas center C has a low number of daycare treatments (ratio 0.3) in relation to their inpatients visits compared to the other centers.

**Fig. 3:**
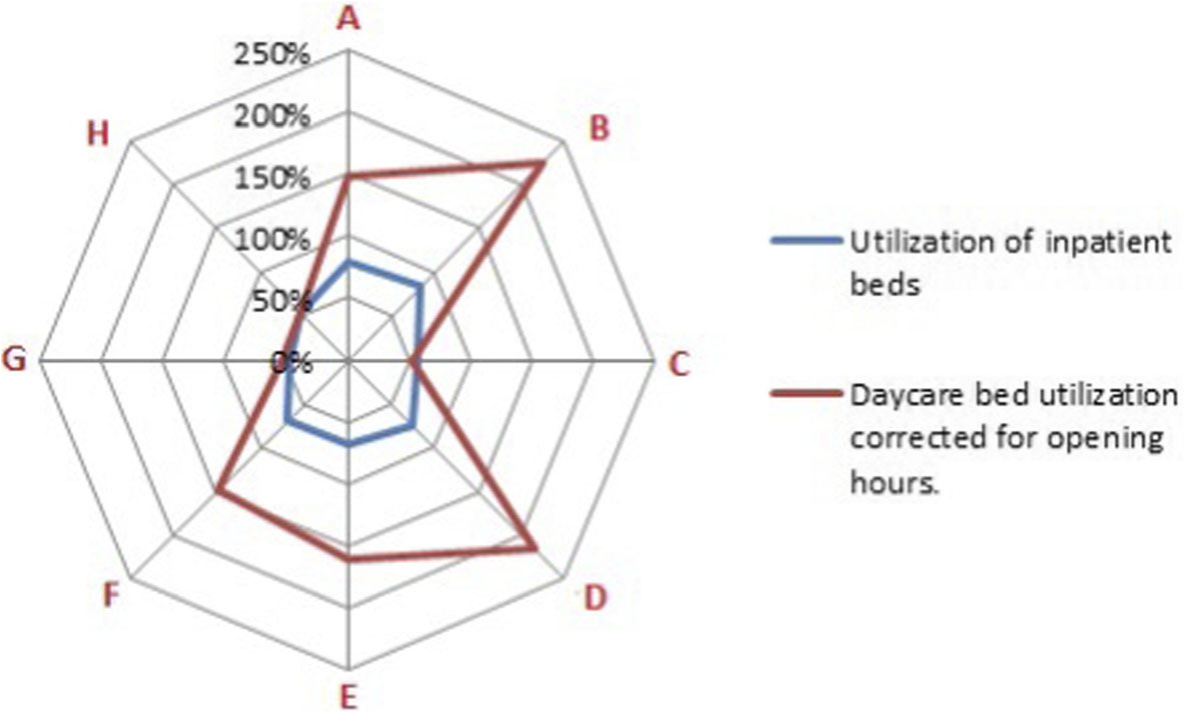
Inpatient and day-care bed utilization

The utilization of beds differs between centers, as shown in Fig. 3. Especially center C, G and H have a relatively low inpatient bed utilization. Similarly, a large variation in utilization of the daycare beds is observed. Center E has a high daycare bed utilization, but scores average in the ratio between daycare treatments/inpatient visits. In contrast, center G also had a relatively high number of daycare treatments but a lower utilization.

##### Input efficiency

**Fig. 4:**
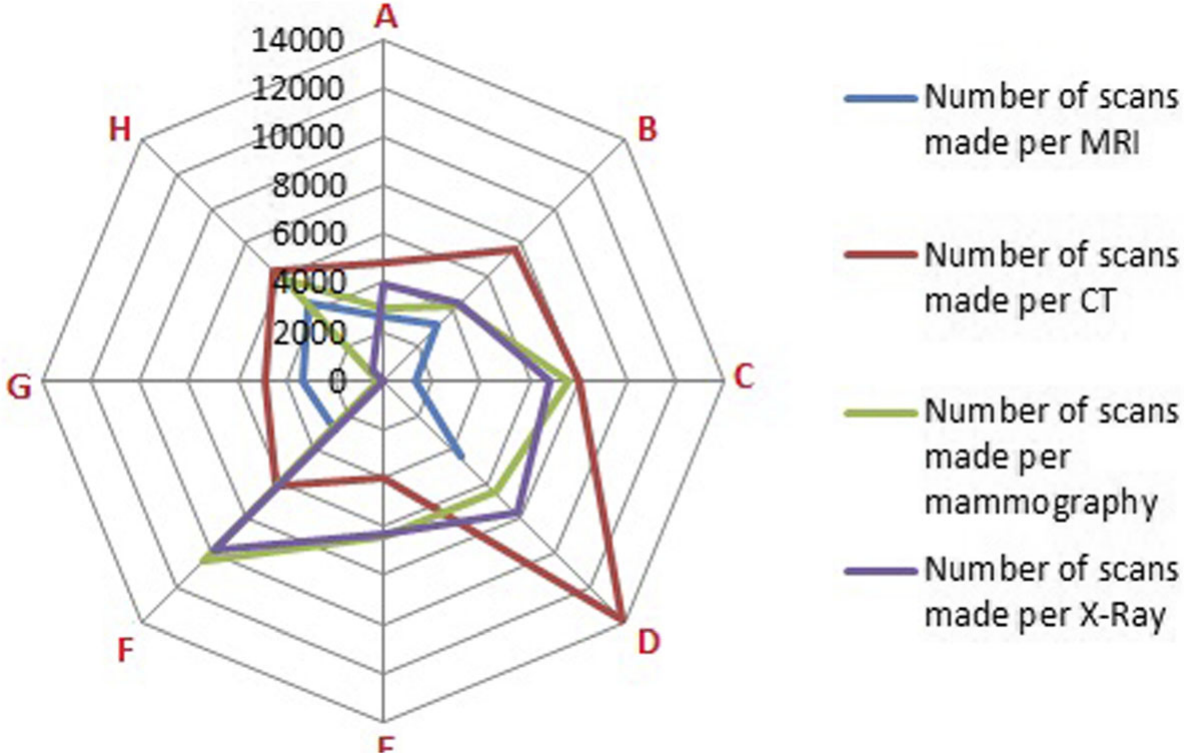
Total number of scans made per device in one year

Number of scans per radiology device varies between centers, as shown in Fig. 4. Center D scores high on the efficiency of MRI (4462 scans per MRI) X-ray (7703 scan per X-ray machine), and CT(13,836 scans). Center H scores high on the efficiency of MRI and CT. Center E has outsourced their MRI and no data was available from center G considering X-rays.

#### Safe

Center A has a safety management system which is audited annually by an independent external agency. Prospective risk assessments are performed in center A before implementing new treatments, new care pathways or critical changes in key processes. Center B divided risk management into general risk management (e.g. risks of fire) and clinical risk management (e.g. transfusion risks and medication errors). Institute H adopted the “International Patient Safety Goals” (IPSG) issued by the Joint Commission International [20]. Most centers (*n* = 7)have an institution-wide reporting systems that registers different types of adverse events: near miss; incident; adverse event; sentinel event. Only doctors can make official notifications of a medical error in institute E and nurses cannot report an incident directly. Center G uses a system that generates reports for patient satisfaction, patient safety and patient complaints. Near misses should be reported in institute H according to their procedures but in practice only actual events are reported. For more information on the domain of safety see Table 3.

#### Patient-centered

Although all center have some type of contact-person for patients, none had an official case-manager for all patient pathways. In institute A and D a formalized inclusion of patients in the strategy development is present. Other centers reported to collaborate with external patient organizations to represent patients. All centers provide some care for cancer survivors, however, only center A has an extensive survivorship program in-house with a dedicated budget. Center G also reports to have a budget for survivorship care (e.g. Psychosocial support). For more information on patient centeredness see Table 3.

#### Timely

For seven centers the waiting times are set by the government (see Table 3). Institute A indicated that they encountered difficulties in meeting the maximum waiting time for some types of surgeries. The maximum waiting times are input for negotiations with healthcare insurers, and have potential influence on the funding for center A. Center H reports waiting times to the regional government who uses this data to adjust the amount of services offered by the regional healthcare-system. Possible reasons mentioned for long waiting times are high demand of patients for diagnostic tests and insufficient staff. The largest variation between institutes occurred in overall waiting time before first visit, which varied between 1.5 and 21.8 days.

### Improvement suggestions

Table 4 describes examples of improvement suggestions per pilot center and resulting improvement plans. Improvement suggestions varied from broader processes such as the involvement of patients in the care process, to specific recommendations (e.g. measure staff satisfaction). Adoption of case managers was a frequently mentioned improvement suggestion. Regarding the suggestion to improve patient participation in the organization, center C only partially agreed as they stated “not all patients want to be involved”. Center A felt a complication registry was mainly useful per discipline and therefore partly agreed with the suggestion to implement an institution-wide complications registry. Out of the total improvement suggestions, pilot centers agreed with 85% and partially agreed with 15%. For center G improvement suggestions were given, however, no improvement plan was received.

**Table 4:** Improvement suggestions, response and planned actions

Suggestions	Institute/Agreement	Comments	Actions to be taken identified by pilot centers
Case managers for (all) patients/all tumor types	A/ Agree	“This is important but requires specialized staff, currently shortage of this specialized staff.”	Currently there are official case managers for 5 tumor types, development of case managers for other tumor types will follow these examples
	B/ Agree	“Case managers are an important tool in patient treatment so we want to improve this area.”	Already part of the strategic vision so no extra actions need to be taken
	C/ Agree	“It would be good to have case manager-the process has to be more organized, more patient oriented.”	Educate the right staff and dedicate them as case-manager
	F/ Agree	“A case manager for each pathway will be formally identified.”	Define clear role and responsibility for the case manager for each pathway/tumor type
Develop more support for survivors	B/ Agree	“With the increase of the survival rates in cancer patients we recognize that this is an area that we must improve.”	A website where survivors can exchange information and experiences was already launched. A portal for survivors, amongst others, is under development.
	D/ Agree	“Survivorship programs are provided mostly by the patient organizations.”	Develop own survivorship program for the institute and further formalize the collaboration with patient organizations in survivorship programs.
Increase patient participation in the care process	B/ Agree	“We are already working in this area.”	An area on the website is under development were patient can access: future appointments, exams results and requisitions, among other clinical and administrative information. The portal that is under development will have one tab containing the patients targeted information.
Improve patient participation in the organization/strategy development	C/ Partially agree	“Patients have to be involved. However not all patients want to be involved.”	All patients have to pass the MDT. And after discussion-take a decision on whether to participate. This participation has to be organized.
Develop a structured, institute wide adverse events analysis system	C/ Agree	“It is absolutely necessary to check and register these events. Important for the quality of care.”	Depends on the staff. Sometimes they hide the information
Measure staff satisfaction	C/ Agree	“Staff has to be honest and not just provide the socially accepted answers.”	Regular discussions with staff, improve existing questionnaires
Central complication registry may be useful	A/ Partially agree	“Complication registration is mainly useful for healthcare professionals, current registration system allows health professionals to see the data important for them, per discipline. Central registration could be useful to annually analyze the results and look at the trends compared to trends in for example new patients. The national institute for Clinical Auditing registers complications as well on a national level.”	Create system that can extract data from existing system or develop new registration system
Implement Computerized Physician Order Entry	E/ Agree		Electronic prescriptions are currently being implemented: in the short term there will be 2 pilot actions for 2 departments. It is currently planned to include treatment details (chemotherapy data), transfusions and clinical trial participation.
	F/ Partially agree	“This is an important and urgent objective, but unfortunately due to regional restrictions the institute cannot be proactively proceed. ”	
Improve patient transition protocol	H/ Agree	“We should improve the network with other hospitals/institutes, care facilities and general practitioners (GPs) as well.”	Improvement of the electronic chart (e-chart): at regional level, the first attempt has been made within the region
Assess and improve inpatient bed utilization	H/ Partially agree	“Inpatient bed utilization is planned and regulated at regional level.”	

## Discussion

In this study, we developed a benchmark tool to assess the quality and effectiveness of comprehensive cancer care consisting of 61 qualitative indicators and 141 quantitative indicators. The tool was successfully tested in eight cancer centers to assess its suitability for yielding improvement suggestions and identifying good practices.

The benchmark data showed performance differences between cancer centers which led to improvement suggestions/opportunities for all participating centers. In general, the indicators revealed well-organized centers. However, there were indicators on which centers performed less. For example, not all centers register mortality rates and it is unclear whether these rates, when registered, are made public. Nevertheless, there is broad consensus that public reporting of provider performance can be an important tool to drive improvements in patient care [21]. An indicator on which only two centers performed well was the offering of in-house survivorship care by having a dedicated budget. An advantage of follow-up taking place in cancer centers is that it is comfortable for patients and provides continuity of care [22]. However, it is debatable whether offering this kind of care should be the responsibility of cancer centers, as multiple pilot centers already indicated to have tight budgets.

Large variety existed in the domain of efficiency between centers. This variety was only partly related to differences in healthcare systems, leading to multiple improvement suggestions. For example, center C, G and H had a relatively low inpatient bed utilization, which is likely to be less cost-efficient. Center G had a high number of daycare treatments but a lower bed utilization, possibly indicating a utilization loss. A higher ratio indicates efficient use of beds and chairs and, hence, most likely also staff use. Centers C and D might have a surplus of daycare beds and chairs. Wind et al. [23] showed that having fewer beds has no association with low financial performance and could indeed improve efficiency.

Another important improvement area was patient-centeredness. Specifically in the area of case management for which all centers agreed that it was necessary to implement or expand. Case management is an organizational approach used to optimize the quality of treatment and care for individuals within complex patient groups [24]. However, centers indicated that implementing or extending these case managers will take a long time and therefore categorized this as mid-term (2–5 years) or long-term (6–10 years) goals.

### Limitations

Several assumptions underpinned this study. First, although we thoroughly searched the literature and existing quality assessments to identify indicators for the initial list, some suitable indicators may have been missed. Identifying suitable outcome indicators was more challenging than for example process indicators due to the difference in case-mix and healthcare system and financing. We tried to minimize this influence by including a large group of experts from various fields who had affinity with development and management of cancer centers and quality assessment in cancer care. We continuously modified the set of indicators in response to feedback on their relevancy, measurability and comparability by the pilot centers. An advantage of this approach is that the indicators benchmark what the cancer centers want to know, which can increase adoption of the benchmark format as a tool for future quality improvement.

Second, the tool was only tested once in eight European cancer centers. This makes it impossible to say whether the benchmark actually led to quality improvements. Consequently, future research should evaluate the implementation of improvement plans to investigate whether the benchmark actually leads to quality improvement. In addition, future inclusion of more centers will allow to assess the actual discriminative capabilities of the indicator set. The benchmark tool was successfully applied in eight European countries with different wealth status. Although differences in healthcare systems and social legislation unavoidably led to differences in nature and availability of data, comparison still revealed relevant and valuable recommendations for all centers. We mainly achieved this by correcting for size, case-mix and type of healthcare reimbursements.

Finally, due to the extensive scope of indicators, it was difficult to go into detail for each topic. A benchmark focused on a single domain would allow to yield more profound information and more specific improvement suggestions and good practices. Future research is therefore advised to focus on specific domains of the BENCH-CAN framework, such as strategy and effectiveness, to gain a more profound understanding of the processes behind the performance differences, enabling a better comparison and more applied improvement recommendations.

### Lessons learned

Multiple lessons were learned from benchmarking cancer care in specialized centers throughout Europe. First, representatives of the pilot centers indicated that international projects such as these can increase awareness that performance can be improved and promote the notion that countries and centers can learn from each other. Identifying successful or good-practice approaches can assist hospitals in improving their services, and reduce inequalities in care provision raising the level of oncologic services across countries. Pilot centers did however indicate not to be able to implement all suggestions or good practices due to socio-economic circumstances. Second, learning through peers enabled cancer centers to improve their performance and efficiency without investing in developing these processes separately. A frequently mentioned comment was the casual, non-competitive atmosphere which led to an open collaboration. Involvement of key stakeholders from the centers at the start of the benchmark is highly recommended to develop interest, strengthen commitment, and ensure sufficient resources which not only accommodates a successful benchmark but also ensures implementation of the lessons learned.

From our earlier review on benchmarking [25], we learned research on benchmarking as a tool to improve hospital processes and quality is limited. The majority of the articles found in this study [25] lacked a structured design, were mostly focused on indicator development and did not report on benchmark outcomes. With this study we used a structured design, reported the benchmark outcomes and contributed to the knowledge base of benchmarking in practice. Although improvement suggestions were made, within the scope of the study we could not report on the effect of the improvement suggestions. This reinforces the need for further research and evidence generation in especially the fields of effectiveness of benchmarking as tool for quality improvement, particularly in terms of patient’s outcomes and learning from good practices.

## Conclusion

In conclusion, we successfully developed and piloted a benchmark tool for cancer centers. This study generated more insight into the process of international benchmarking, providing cancer centers with common definitions, indicators and a tool to focus, compare and elaborate on organizational performance. Results of the benchmark exercise highlight the importance of an accurate description of underlying processes and understanding the rationale behind these processes. The tool allowed comparison of inter-organizational performance in a wide range of domains, and improvement opportunities were identified. The tool and the thereof derived improvement opportunities were positively evaluated by the participating cancer centers. Our tool enables cancer centers to improve on quality and efficiency by learning from good practices from their peers instead of reinventing the wheel.

## Additional files

Additional file 1: **Appendix 1**. Semi-structured interview topic list. This file contains some examples of topics that were discussed during the semi-structured interviews. (PDF 134 kb)

Additional file 2: **Appendix 2A.** Qualitative indicators. This file contains the qualitative indicators that were used in the benchmark. **Appendix 2B.** Quantitative indicators. This file contains the quantitative indicators that were used in the benchmark. (ZIP 1350 kb)
