# Supplementary material for: Development of a benchmark tool for cancer centers; results from a pilot exercise
Source: BMC Health Serv Res. 2018 Oct 10;18:764. doi: 10.1186/s12913-018-3574-z (PMC6180585; doi:10.1186/s12913-018-3574-z)
Supplement: Supplementary file 1 — Appendix 1. Semi-structured interview topic list. This file contains some examples of topics that were discussed during the semi-structured interviews. (PDF 134 kb) [file 12913_2018_3574_MOESM1_ESM.pdf]

Appendix 1 Sample of BENCH-CAN semi-structured interview topic list and questions

1. Quality Control

Indicator 3.2a Strategies/systems for quality improvement

Questions: - You say that the quality manager sets the goals for quality management and that this is audited externally. How does this work?

- QC referents, do they have a medical background? Are they fulltime available for quality control?

Indicator 3.4a Adverse event analysis

Questions: - Adverse event / near misses system. Please explain how this works

- What is included?
- Who are allowed to notify?
- Is everybody notifying?
- – Can a notifier see what is happening with the notification?
- – What is done with the notification (also frequency)?
- – Please describe a top 3 most identified items
- Within what timeframe irregularities investigated?
- Where do employees get the forms or is notification digitally?

Indicator 3.4b Results adverse events analysis

Questions: Are patient complaints anonymous? Is the care giver named in the complaint?

Indicator 8.1 Incidents with hazardous materials and products 2012

Questions: - What kind of incidents were these?

2. HR

Indicator 2.2a Types of education that are offered by your institute (in house)

Questions: - Could you please describe which courses are offered inhouse and which external?

- Is there a set budget for following external education?
- Who approves the requests for external education?
- Is there a policy to exchane information from courses?

Indicator 2.2b Education-needs analysis

Questions: - Is there a link with requirements for the accreditation for professionals by

external regulatory bodies
- Could we see the form?

Indicator 2.2c Staff training on quality and risk management is provided to all staff
Questions: - What is taught within the quality assurance training?

Indicator 2.4 Sick-leave registration
Questions: - Is this information communicated to the departments?
- Is it discussed in meetings?

3. IT

Indicator 4.3a The institute has a digital system (that stores diagnostic, treatment and outcome data etc. on each patient)
Questions: - Please draw a figure that shows how the systems are related to each other
- Please describe wheter the systems are standard packages or have been developed by/for the Institute?
– Number of staff working in IT department

4. Head of Research (or Head of a Research Department)

Indicator 3.1b Research portfolio
Questions: - How is decided on which tumors to focus?
- Can the institute decide by themselves how much money to spend on each research program or is this mainly set by external parties?

Questions: - Could you please describe the organizational structure of research at your center?
- Research groups(PI, MD, non MD, PhD's)
- Supportive services (Patent office etc.)

5. Head of Clinical Department (or Head Physician/Nurse)

Indicator 3.3b medication management

Questions: - Is prescription of chemotherapy/opiats checked by 2 physicians?

– Before administering the chemotherapy do 1 or 2 nurses check the prescription?

Indicator 4.2c Transition protocol

Questions: - Are there rules for patients discharge?

- If the patient goes to another care facility how is the communication with this other facility organized or is this done by the patient himself?

Indicator 5.2 Guideline access

Questions - How are guideline translated to daily practice use(protocols etc.)?

- If there is some deviation from the guideline is this registered?
- What is done with the registration mentioned above?
- US or EU guidelines?

Indicator 5.3 Ensuring patient safety

Questions: - Who performs the audits?

- On which basis are operational procedures updated?

Indicator 5.5 follow up system

Questions: - Organized by specialty or tumour type?

- Included in guidelines?
