# Supplementary material for: Development of a benchmark tool for cancer centers; results from a pilot exercise
Source: BMC Health Serv Res. 2018 Oct 10;18:764. doi: 10.1186/s12913-018-3574-z (PMC6180585; doi:10.1186/s12913-018-3574-z)
Supplement: Supplementary file 2 — Appendix 2A. Qualitative indicators. This file contains the qualitative indicators that were used in the benchmark. Appendix 2B. Quantitative indicators. This file contains the quantitative indicators that were used in the benchmark. (ZIP 1350 kb) [file 12913_2018_3574_MOESM2_ESM.zip › appendix 2A and B/Appendix 2A Qualitative indicatorsR2.pdf]

1. Leadership

1.1 Organization	**Indicator 1.1a: Organogram**<br><br>Description: Provide the organogram of your institute.<br>This indicator will not be used for benchmarking but serves as background information.
1.2 Communication	**Indicator 1.2b: Communication with other parties**<br><br>Description: Please describe how the board of directors communicates with other parties.<br><br>Definition: other parties include: patient representatives, clinical department heads, research department heads, government (ministry of health). Communication strategies could include meetings, phone calls, and emails.<br><br>Measurement: Give an overview of communication strategies and channels and please provide documentation and information about frequency.<br><br>Performance level: High administrative level.

2. People

2.1 Staff turnover	**Indicator 2.1a: Yearly turnover rate**<br><br>Description: Describe the yearly staff turnover rate at the institute. If staff turnover is due to governmental regulations, please indicate.<br><br>Numerator: Number of staff leaving in year X<br><br>Denominator: Average number of employees in year X<br><br>Definition: Employee turnover refers to the rate at which employees leave jobs in a company, this excludes leaving because the end of a (training) contract is reached.<br><br>Measurement: Numerator/Denominator<br><br>Performance level: Institutional
	**Indicator 2.1b Voluntary termination of contract and average length of contract**<br><br>Description: Please describe for nurses in the clinical departments how many ended their contract based on their own initiative in the year X and the average lengths of time those people were working at the institute.<br><br>Numerator 1: Number of nurses ending their contract on their own initiative in the year X<br><br>Denominator 1: Total number of nurses working in clinical departments the year X<br><br>Numerator 2: Total years of work of leaving nurses<br><br>Denominator 2: Total number of leaving nurses<br><br>Measurement: Numerator/denominator<br><br>Performance level: Clinical department
	**Indicator 2.1c Voluntary termination of contract and average length of contract**<br><br>Description: Please describe for physicians in the clinical departments how many ended their contract based on their own initiative in the year X and the average of how long those people were working at the institute.

	<u>Numerator 1:</u> Number of physicians ending their contract on their own initiative in the year X<br><br><u>Denominator 1:</u> Total number of physicians working in clinical departments the year X<br><br><u>Numerator 2:</u> Total years of work of leaving physicians<br><br><u>Denominator 2:</u> Total number of leaving physicians<br><br><u>Measurement:</u> Numerator/denominator<br><br><u>Performance level:</u> Clinical department
	**Indicator 2.1d Exit interviews**<br><br><u>Description</u>: Does the institute have exit interviews with exiting staff?<br><br><u>Definition</u>: Exiting staff is staff terminating their contract and thus leaving the institute. It excludes people that are leaving because their (training) contract ended.<br><br><u>Measurement</u>: Yes; mostly; partially; no; not applicable (If yes, please indicate which personnel is responsible for conducting the exit interviews)<br><br><u>Performance level</u>: Institutional, if applicable on department level
	**Indicator 2.1e: Information exit interviews**<br><br><u>Description</u>: Is the information gathered through the exit interviews used for performance improvement?<br><br><u>Definition</u>: Exit interview is the conversation that's being held when the staff member hears the contract is ended preliminary, or after the contract is ended.<br><br><u>Measurement</u>: Yes; mostly; partially; no; not applicable (If yes, please indicate how)<br><br><u>Performance level</u>: Institutional, if applicable on department level
2.2 Staff training	**Indicator 2.2a: Types of education offered in-house**<br><br><u>Description:</u> This indicator assess the education at the institute: e.g. how many courses are offered? What kinds of courses are offered? (multidisciplinary educations) To whom are the courses offered? Is it only for staff or outsiders as well? Credit points? Evaluation sheets? etc…

	Definition: A credit point relates to the point for the course being officially accredited by a university or other institution other than the cancer institute.<br><br>Measurement: Give an overview of the items listed above; please divide the types of education by stakeholder: e.g. do you have courses for bachelor and/or master students, PhD students, physicians, nurses, administrative personal or others. Indicate for each course if credit point can be received and whether other people can attend.<br><br>Performance level: Institutional level
	**Indicator 2.2 b: Education needs analysis**<br><br>Description: Does the institute have a system for education needs analysis?<br><br>Definition: System includes any kind of ICT based or in person based assessment.<br><br>Measurement: Yes/no, yes explain if so, how often are the needs analysed? Please indicate whether there is a link with professional accreditation bodies (physician or nurse accreditation).<br><br>Performance level: System for all employees that need to keep their knowledge up to date; physicians, researchers, nurses.
	**Indicator 2.2c: Staff training**<br><br>Description: Is training on quality and risk management provided to all staff?<br><br>Definition: Training could mean a day long course or any other type of training. Staff means everybody with a contract at the institute.<br><br>Measurement: Is it provided? (yes/no) What is being taught? And how often is it provided?<br><br>Performance level: Institutional
2.3 Recruitment	**Indicator 2.3a: Responses to vacancies**<br><br>Description: Describe the average number of responses per vacancy in the year X. Please also describe how vacancies are advertised.<br><br>Numerator: Responses to vacancies the year X

	Denominator: Number of vacancies the year X<br><br>Definition: A vacancy is a position that is unfilled and for which the institute is looking for a new employee.<br><br>Measurement: Numerator/Denominator<br><br>Performance level: Institutional, data probably provided by HR-department

3. Strategy

3.1 Focus	**Indicator 3.1a: Focus on tumour type- care**<br><br>Description: Does the centre have a focus on certain tumour types in terms of treatment?<br><br>Definition: A tumour type is a tumour in area of the body, not a specific tumour, so cancer in the breast not a ductal carcinoma in situ.<br><br>Measurement: Yes/no, if yes explain<br><br>Performance level: Institutional
	**Indicator 3.1b: Focus on tumour type- research**<br><br>Description: Does the centre have a focus on certain tumour types in terms of research?<br><br>Definition: A tumour type is a tumour in area of the body, not a specific tumour, so cancer in the breast not a DCIS.<br><br>Measurement: Yes/no, if yes explain<br><br>Performance level: Institutional
	**Indicator 3.1c: Organizational structure of research**<br><br>Description: Could you please describe the organizational structure of research at your centre?<br><br>Measurement: organizational structure relates to the way the research departments are organized. Is there for example one PI and several post-docs and PhD's for each research groups or is research organized per department. Please also describe if there are any supportive facilities for doing research for example a patent office or a dedicated person to help with grant applications.<br><br>Performance level: Institutional
	**Indicator 3.1d: Top 3 most common tumours**<br><br>Description: Describe the 3 most common tumour types treated in the year X.<br><br>Definition: A tumour type is for example breast cancer, all types

	Measurement: Most common; Second; Third.<br><br>Performance level: Institutional<br>This indicator will not be used for benchmarking but serves as background information.
3.2 Quality improvement	**Indicator 3.2a: Strategies/systems for quality improvement**<br><br>Description: Describe strategies used for quality improvement (logistics, research, education, multidisciplinary teams, etc.) as listed in the year/multi year plan, if applicable.<br><br>Measurement: List all quality improvement strategies/systems and describe them for the year X. Please include both external systems (accreditation for example OECI) and internal systems and quality improvement strategies based on internal evaluations. Please make a distinction between improvement strategies for care, research, the whole institute and if applicable education.<br><br>Performance level: Institutional
	**Indicator 3.2b: Measurable goals**<br><br>Description: Does the institute set out measurable goals for the quality improvement strategies? Please provide examples.<br><br>Definition: Quality improvement strategies are the strategies described in the previous indicator.<br><br>Measurement: Indicate for each strategy the goals that were set beforehand to evaluate the effectiveness of the strategy and, if possible, indicate the results of the strategies based on the goals for the year X.<br><br>Performance level: Institutional
3.3 Risk and safety management	**Indicator 3.3a: Risk and safety management**<br><br>Description: Are there strategies for risk and safety management? If so please describe these strategies.<br><br>Definition: Risk management includes for example protocols for staff that work with biological/chemical hazards, waste management, evaluation of contamination risks etc…<br><br>Measurement: Description of the different strategies used.<br><br>Performance level: Institutional

	**Indicator 3.3b: Medication management**<br><br>Description: How are drugs given, stored and registered/followed in inventory? How is it ensured that drugs are given to the right person?<br><br>Measurement: Description of how drugs are given stored and registered/followed in inventory. How is it ensured that drugs are given to the right person?<br><br>Performance level: Institutional
3.4 Adverse events	**Indicator 3.4a: Adverse event analysis**<br><br>Description: Is there a program for systemic analysis of major adverse or undesirable events? If so please describe.<br><br>Definition: Adverse events are events that occur in the treatment of patients and that are undesired and have a negative impact. This could be caused by a medical error for example. examples of notifications are:<br><br>Near misses - Risk Factor or potential error that is intercepted before the event occurs or causes injury,<br><br>Incident - Unexpected or unintended event that, or has caused or will cause damage to the patient,<br><br>Adverse event -Unintentional injury or complication which results in disability, hospitalization prolongation or patient's death, as a consequence of healthcare provided,<br><br>Sentinel event - Adverse event subtype that is rare but extremely serious.<br><br>Measurement: Description of the analysis program, what does it measure (which events are included)? Who are allowed to notify? Can a notifier see what happened with the notification?<br><br>Performance level: Institutional
	**Indicator 3.4b: Results adverse events analysis**<br><br>Description: Are the results of the adverse events program analysed? What is done with the results? Are they used for quality improvement strategies? If so how?<br><br>Measurement: Are the results analysed yes/no? What is done

	with results, are the made public for example? Please describe.<br><br><u>Performance level</u>: Institutional

4. Partnerships and resources

4.1 Cooperation with universities	**Indicator 4.1a: Description of cooperation agreements with universities**<br><br>Description: Describe if and how the institutes cooperates with universities (in terms of PhD students, joint clinical/translational research projects etc).<br><br>Measurement: If applicable, description of how cooperation is organised/documented and with whom (which universities).<br><br>Performance level: Institutional, if applicable only for the research department.
	**Indicator 4.1b: Number of physicians with appointments (contracts) at universities / professorships**<br><br>Description: How many physicians that are currently working as at the institute have a contract with a university as well?<br><br>Definition: An appointment (contract) means working for the university as well, not a one-time collaboration.<br><br>Measurement: Give the number of physicians that are currently working as a physician at the institute and a university.<br><br>Performance level: Institutional
4.2 Cooperation with external partners	**Indicator 4.2a: Organization of collaboration with other institutes/care providers**<br><br>Description: Network (local, regional, national, other) Communication (electronic file sharing, e-mail, phone, joint meetings)<br><br>Definition: Other institutes could be other cancer institutes or any other care facility<br><br>Measurement: Describe with whom there is collaboration and how this collaboration is organised. Describe the structure of the collaborating activities, potential treaties, agreements.<br><br>Performance level: Institutional
	**Indicator 4.2b: External partners**<br><br>Description: Describe the main external partners of the

	institute (research institutes, screening facilities etc.).<br><br>Definition: External partners are all partners that provide services needed by the institute, which are not part of the institute.<br><br>Measurement: List all partners.<br><br>Performance level: Institutional
	**Indicator 4.2c: Transition protocol**<br><br>Description: Describe, if applicable, the protocol for the transfer of patients to other facilities.<br><br>Definition: Patients can be any patient that was treated by the institute but will no longer be and is being transferred to another facility (for example a hospice).<br><br>Measurement: Describe how the transition is organised. This could be for example by providing the patients a discharge letter. Please also provide documentation.<br><br>Performance level: Institutional
4.3 ICT	**Indicator 4.3a Electronic patient record (EPR)**<br><br>Description: Please describe the ICT (Information and Communication Technology) system used at your institute in terms of EPR (Electronic patient record).<br><br>Definitions: An ICT system is any computer or mobile device-based system, so no paper-based system. An EPR, also referred to sometimes at Electronic Health Record is a tool to view a patient’s medical record via a computerised interface. Examples of data that can be stored in an EPR are:<br><br>• Vital patient functions (blood pressure, temperature)<br><br>• Diagnosis and treatment plans<br><br>• Summary of outpatient visits<br><br>Measurement: Give a description of the system and for which purposes it is used. If different ICT systems are in place for this purpose, please describe all of them. Please also indicate what kind of data is being stored, for how long, who is handling the system and how access is granted.<br><br>Performance level: Institutional, if applicable departmental

	**Indicator 4.3b Computerized physician order entry (CPOE)**<br><br>Description: Please describe the ICT system used at your institute in terms of a CPOE.<br><br>Definitions: A CPOE is a process of electronic entry of medical practitioner instructions for the treatment of patients (particularly hospitalised patients) under his or her care. These orders are communicated over a computer network to the medical staff or to the departments (pharmacy, laboratory, or radiology) responsible for fulfilling the order.<br><br>Measurement: Give a description of the system and for which purposes it is used. If different ICT systems are in place for this purpose please describe all of them. Please also indicate what kind of data is being stored, for how long, who is handling the system and how access is granted.<br><br>Performance level: Institutional, if applicable departmental
	**Indicator 4.3c ICT support research**<br><br>Description: Please describe the ICT system used at your institute for research purposes and how ICT supports research.<br><br>Definitions: An ICT system is any computer or mobile device-based system, so no paper-based system. A research system could be for example a database.<br><br>Measurement: Give a description of the system and for which purposes it is used, who or what assists researchers. If different ICT systems are in place for this purpose please describe all of them. Please also indicate what kind of data is being stored, for how long, who is handling the system and how access is granted.<br><br>Performance level: Institutional, if applicable departmental
	**Indicator 4.3d External exchange**<br><br>Description: Please describe if it is possible to share data from your institute with external parties such as other hospitals or care facilities.<br><br>Measurement: Give a description of the possibilities to share information with external parties. If so, what kind of information (for example data from the EPR). Please describe with whom this data is shared.

	Performance level: Institutional, if applicable departmental

5. Processes, products and services

5.1 Patient centred	**Indicator 5.1a: Case managers**<br><br>Description: Is there one staff member appointed as a contact person or “case manager” for each patient?<br><br>Definition: The contact person is the central source of information for the patient; this could be a nurse, a physician, a social worker or other member of staff. Person needs to be under contract with the institute.<br><br>Measurement: Is there a contact person for each patient? If not for which percentage of the patients is there a contact person? What is the most common background of the case manager (e.g. is it often a nurse, a physician etc.)?<br><br>Performance level: Institutional
	**Indicator 5.1b: Patients’ participation in the diagnostic and treatment process**<br><br>Description: Please describe which options are given to patients to participate in their diagnostic and treatment process for example by having insight in their own treatment plan and health data.<br><br>Definitions: Patients are all people treated at the institute both in-hospitals as in the polyclinic.<br><br>Measurement: Description of the options<br><br>Performance level: Institutional
	**Indicator 5.1c: Patients’ participation in strategy development**<br><br>Description: Please describe whether patients can participate in the strategy development of the institute<br><br>Definitions: Patients are all people treated at the institute both in-hospital as in the polyclinic.<br><br>Measurement: Describe if patients can participate (yes/no). If so, how do they participate?<br><br>Performance level: Institutional
	**Indicator 5.1d: Patients’ education**

	Description: Does the institute provide education to patients<br><br>Definitions: Types of education could include for example: “Everything you need to know about chemotherapy and treating its side effects”; courses on diets for cancer patients; Preparing for operation – both body and soul.<br><br>Measurement: Yes/no, yes explain if so what kind of education on which topics is provided.<br><br>Performance level: Institutional level, if applicable per department
	**Indicator 5.1e: Patients reminders**<br><br>Description: Please describe, if applicable, if and how the patients are reminded that they have a visit to the hospital coming up. If this is only in place for certain departments please indicate.<br><br>Definitions: Reminders can be a mobile texts or an e-mail, for example.<br><br>Measurement: Describe if patients receive reminders and how they receive these reminders.<br><br>Performance level: Institutional
5.2 Guidelines	**Indicator 5.2a: Guideline access**<br><br>Description: How are guidelines accessed and stored within the institute? Are the guidelines updated and controlled by experts on a regular basis, if so by whom and how often?<br><br>Definitions: A guideline is an indication of policy or procedure by which to determine a course of action.<br><br>Measurement: Describe the system used to store and manage guidelines within the institute (ICT, paper based, other), are the guidelines updated and by whom? Are the guidelines based on US or EU guidelines?<br><br>Performance level: Institutional/per department.
	**Indicator 5.2b Guideline to protocol**<br><br>Description: How are guidelines translated into protocols for daily use?<br><br>Definitions: A guideline is an indication of policy or procedure

	by which to determine a course of action. A protocol (also referred to as a standard operating procedure) is a locally agreed standard to which clinicians and the organization can work and against which they can be audited.<br><br>Measurement: Describe how protocols are developed at the institute, who develops them and how often they are updated. How is it checked if all procedures are done according to the protocol and what happens if there is a deviation from the protocol?<br><br>Performance level: Institutional/per department.
5.3 Patient safety	**Indicator 5.3a: Ensuring patient safety**<br><br>Description: Please describe how, if applicable, patient safety is ensured at the institute.<br><br>Definitions: Patient safety is the prevention of errors and adverse effects to patients associated with health care.<br><br>Measurement: Please list which strategies or systems are used to ensure patient safety and which indicators are being measured. For example infections, ISO standards etc. Please describe whether these are mandatory by the government or other regulatory agency.<br><br>Performance level: Institutional
5.4 Follow up	**Indicator 5.4a: Follow-up system**<br><br>Description: Please describe how the follow-up is organised at the institute<br><br>Definition: Follow-up includes monitoring a person's health over time after treatment. This is usually done by regular medical check-ups. The frequency of these check-up could vary per patient, institute and type of cancer.<br><br>Measurement: Describe how follow-up care is organised: e.g. is it performed by the institute itself or others? How are appointments scheduled? Is follow-up organised by specialty (surgery, radiotherapy) or by tumour type? Is follow-up included in guidelines?<br><br>Performance level: Institutional
5.5 Survivorship	**Indicator 5.5a: Description of support**<br><br>Description: Please describe, if applicable what kind of support

	is offered by the institute to survivors.<br><br>Definitions: A survivor is a patient that has completed initial cancer management.<br><br>Measurement: Describe all kinds of support that are provided by the centre itself, so not by others outside the institute.<br><br>Performance level: Institutional

6. Effective

6.1 Mortality rates	**Indicator 6.1a: Types of mortality rates**<br><br>Description: Please describe the types of mortality rates that your institute can provide.<br><br>Definition: Mortality rate is the ratio of deaths.<br><br>Measurement: What kind of mortality rates can you provide and please provide them for year X?<br><br>Performance level: Institutional
	**Indicator 6.1b: Colorectal surgery mortality**<br><br>Description: Proportion of in-hospital mortality within 30 days after colon or rectal cancer surgery (for non-urgent surgery).<br><br>Numerator: Patients that died within 30 days in year X<br><br>Denominator: Total number of patients treated for given tumour in the year X<br><br>Definition: Only pre-planned surgeries should be counted, no urgent surgeries.<br><br>Measurement: Numerator/Denominator<br><br>Performance level: Institutional (data from responsible department/mortality registry)
	**Indicator 6.1c: Breast surgery mortality**<br><br>Description: Proportion of in-hospital mortality within 30 days after breast cancer surgery (for non-urgent surgery).<br><br>Numerator: Patients that died within 30 days in year X<br><br>Denominator: Total number of patients treated for given tumour in the year X<br><br>Definition: Only pre-planned surgeries should be counted, no urgent surgeries.<br><br>Measurement: Numerator/Denominator<br><br>Performance level: Institutional (data from responsible department/mortality registry)

6.2 Complication rates	**Indicator 6.2a: Complication rates registration**<br><br>Description: Are complication rates registered? If so which complication rates are registered and where or how are they registered?<br><br>Definition: Complication rates contain e.g. the Clavien rate for surgery or toxicities for chemotherapy. Complications can be registered in for example the patients file, in a central location etc…<br><br>Measurement: Are complication rates measured (yes/no)? If so which ones are measured? How/where are they registered?<br><br>Performance level: Institutional, if applicable per department
	**Indicator 6.2b: Complication rates**<br><br>Description: Please provide data on the above described complication rates, if applicable. What is done with the information from the complication rates?<br><br>Measurement: Are complication rates measured? Provide data on these rates. Please describe how this information is used within the institute, for example for quality improvement.<br><br>Performance level: Institutional, if applicable per department

7. Safe

7.1 Work- safety	**Indicator 7.1a: Incidents with hazardous materials and products**<br><br>Description: Please describe the number of incidents with hazardous material in the year X.<br><br>Numerator: Number of incidents with hazardous material in year x<br><br>Denominator: Total number of employees that worked with hazardous materials year x<br><br>Definition: Hazardous material is any item or agent (biological, chemical, physical) which has the potential to cause harm to humans, animals, or the environment, either by itself or through interaction with other factors. An incident is an occurrence or event that interrupts normal procedure or harms a human, animal or the environment.<br><br>Measurement: Number of incidents in the year X.<br><br>Performance level: Departmental
7.2 Patient safety	**Indicator 7.2a: Patient safety monitoring**<br><br>Description: Please indicate the number of incidents with patient safety in the year X.<br><br>Definition: Patient safety is the prevention of errors and adverse effects to patients associated with health care.<br><br>Measurement: Please describe if monitored, the number of patient safety incident and list the top three of most common incidents.<br><br>Performance level: Institutional
	**Indicator 7.2b: Patient safety indicators**<br><br>Description: Please describe, if applicable, which indicators are measured regarding patient safety in the institute<br><br>Definitions: Patient safety is the prevention of errors and adverse effects to patients associated with health care; indicators are measures that give an indication of output quality or give an indication of process quality.<br><br>Measurement: Please list all patient safety indicators that are

	measured in the institute<br><br><u>Performance level:</u> Institutional
The following indicators are examples of patient safety indicators. Please provide data for these indicators if possible, if not, continue to domain 8.	
7.3 Patient safety (surgeries)	**Indicator 7.3a: Number of surgeries per year**<br><br><u>Description</u>: Is there a minimum of surgeries that need to be performed per year?<br><br><u>Measurement</u>: Please indicate if there is a set minimum of surgeries that have to be performed per year (this is usually done in order to ensure quality of the surgeries) and by whom this minimum is set (e.g. government, associations of medical professionals, other).<br><br><u>Performance level</u>: Surgical department
If the answer to previous question was no, please continue to 7.4	
7.3 Patient safety (surgeries)	**Indicator 7.3b: Number of surgeries for resection of the colon in colorectal cancer patients per year**<br><br><u>Description</u>: What is the minimum amount of colon resections that need to be performed per year (if applicable)? Did you manage to perform enough surgeries based on the norm in the year X?<br><br><u>Definition</u>: A colon resection is a surgical procedure in which all or part of the colon is resected.<br><br><u>Measurement</u>: Please indicate the number of surgeries that have to be performed per year (this is usually done in order to ensure quality of the surgeries) and if you managed to perform this amount in the year X.<br><br><u>Performance level</u>: Surgical department
	**Indicator 7.3c: Number of skin-sparing mastectomies in breast cancer patients per year**<br><br><u>Description</u>: What is the minimum amount of skin-sparing mastectomies that need to be performed per year (if applicable)? Did you manage to perform enough surgeries based on the norm in the year X?

	Definition: Mastectomy is the surgery in which the entire breast in removed. With a skin-sparing mastectomy most of the skin over the breast (other than the nipple and areola) is left intact.<br><br>Measurement: Please indicate the number of surgeries that have to be performed per year (this is usually done in order to ensure quality of the surgeries) and if you managed to perform this amount in the year X.<br><br>Performance level: Surgical department
7.4 Patient safety (sepsis and pressure ulcers)	**Indicator 7.4a: Sepsis after the insertion of a drip-feed into the vena cava superior or vena cava inferior**<br><br>Description: Please indicate the number of cases of sepsis after the insertion of a drip into the great vein close to the heart per 1000 catheter days in the year X.<br><br>Numerator: Cases of sepsis after the insertion of a drip into the great vein close to the heart<br><br>Denominator: 1000 catheter days<br><br>Definition: Catheter Days are days when drip-feeds are inserted in patients. A drip-feed is a device for introducing fluid drop by drop into a patient.<br><br>Measurement: Numerator/Denominator<br><br>Performance level: Applicable departments
	**Indicator 7.4b: Percentage of patients who get pressure ulcers during their stay in hospital**<br><br>Description: Percentage of patients who get pressure ulcers during their stay in hospital<br><br>Numerator: Number of patients that gets pressure ulcers during their stay in hospital in the year X<br><br>Denominator: All patients staying at least two days in the hospital in the year X<br><br>Definition: Pressure ulcers -also called bedsores- are injuries to skin and underlying tissue resulting from prolonged pressure on the skin (ulcers can occur when you are sitting or lying in the same position for a long time).<br><br>Measurement: Numerator/Denominator

	Performance level: Applicable departments

8. Responsive and personalized

8.1 Patient satisfaction survey	**Indicator 8.1a: Patient satisfaction survey**<br><br>Description: Does the institute have a pathway patient satisfaction survey? If so how often is this survey conducted?<br><br>Measurement: Survey, yes/no? How often is it performed? What is done with the results?<br><br>Performance level: Institutional, or per department if applicable

The European Cancer Consumer Quality Index developed for the BENCH-CAN project; the ECCQI can be used to measure the rest of the domain of Responsive and personalized (See Annex 3).

9. Integrated care

9.1 Multidisciplinary teams	**Indicator 9.1a: Composition of multidisciplinary teams**<br><br>Description: How is the participation of personnel in multidisciplinary teams decided? Which protocols are used?<br><br>Definition: Multidisciplinary teams are teams consisting of different professionals with different backgrounds that together discuss patients and decide on treatment plans.<br><br>Measurement: Describe how it is decided which disciplines are in which multidisciplinary team. Is this decided based on general guidelines?<br><br>Performance level: Institutional, if applicable departmental
	**Indicator 9.1b: Patients treated by multidisciplinary teams**<br><br>Description: Are all patients treated by multidisciplinary teams? If not how is it decided who is and who isn't?<br><br>Definition: Multidisciplinary teams are teams consisting of different professionals with different backgrounds that together discuss patients and decide on treatment plans.<br><br>Measurement: Describe if all patients are treated by multidisciplinary teams. If not please describe the patient selection criteria.<br><br>Performance level: Institutional, if applicable departmental
9.2 Research-care integration	**Indicator 9.2a: Research-care**<br><br>Description: If applicable, how is the research department connected to the patient care departments?<br><br>Measurement: Describe how the research department is connected to the patient care departments for example by a department of translation research, physicians doing research, research results translated from bench to bedside.<br><br>Performance level: Institutional, if applicable departmental

10. Timely

10.1 Waiting and throughput times registration	**Indicator 10.1a: Waiting and throughput times registry**<br><br>Description: Please describe if there are procedures for the recording of waiting and throughput times, are there maximum waiting and throughput time, are they set by the institute or for example by the government<br><br>Definitions: Waiting time is the time a patient has to wait for example between referral by a GP and the first visit to the institute.<br><br>Measurement: Describe if waiting and throughput times are recorded, if there are set maximum times, is the information gathered used for improvement and how.<br><br>Performance level: Institutional, if applicable departmental
10.2 Waiting and throughput times	**Indicator 10.2a: Waiting time first visit to institute**<br><br>Description: Describe the average waiting time between the referral and the first visit to the institute.<br><br>Definition: Waiting time between referral by for example a GP and the first visit to the institute.<br><br>Measurement: Describe the average waiting time in the year X.<br><br>Performance level: Institutional, if applicable departmental
	**Indicator 10.2b: Average waiting time between first visit and diagnosis**<br><br>Description: Describe the average waiting time between the first visit at the institute and the appointment in which the diagnosis is discussed.<br><br>Measurement: Describe the average waiting time in the year X.<br><br>Performance level: Institutional, if applicable departmental
	**Indicator 10.2c: Average waiting time between diagnosis and establishing the treatment plan**<br><br>Description: Describe the average waiting time between the appointment in which the diagnosis is discussed and the appointment in which the treatment plan is

	discussed/established.<br><br>Measurement: Describe the average waiting time in the year X.<br><br>Performance level: Institutional, if applicable departmental
	**Indicator 10.2d: Average waiting time between establishing treatment plan and first treatment**<br><br>Description: Describe the average waiting time between the appointment in which the treatment plan is discussed/established and the first treatment.<br><br>Measurement: Describe the average waiting time in the year X.<br><br>Performance level: Institutional, if applicable departmental
